# Correspondence on “Pesticide Residues in Organic and Conventional Agricultural Soils across Europe: Measured and Predicted Concentrations”

**DOI:** 10.1021/acs.est.5c02504

**Published:** 2025-07-03

**Authors:** Albrecht Benzing

**Affiliations:** † Ecological Plant Protection, University of Kassel, Nordbahnhofstrasse 1a, 37213 Witzenhausen, Germany

Knuth et al.^1^ have gathered a highly valuable data set from 10 European countries, in which they compare pesticide residues in soils from 101 organic farms to 100 conventional farms. This kind of data did not exist before, and it confirms results from similar smaller previous studies, especially in terms of real maximum half-lives of some pesticides in soil being much longer than those reported, e.g., in PPDB.^2^

What is especially surprising is the high frequency of pesticide detection in soils from organic farms, combined with high residue concentrations in *some* of these soil samples. The authors confirm that all of the organic farms had started organic management at least 5 years before the soil samples were taken. Looking at the raw data published as Supporting Information, the aggregated mean pesticide load per soil sample is 184 μg kg^–1^ for organic farms, i.e., 8 times lower than for conventional farms (1481 μg kg^–1^). The data set covers current-use pesticides (CUPs) as well as a number of pesticides no longer approved in the EU. Given that EU law establishes a grace period of 6 months for sales, and another 12 months for using up existing stock by farmers,^3^ we can consider all substances as “non-approved”, for which approval had expired before July 1, 2019, i.e., 18 months before starting sampling on January 1, 2021. When we exclude these non-approved pesticides, as well as substances allowed in organic farming (pyrethrin and spinosyn), then we are looking only at synthetic CUPs, which are allowed in conventional farming but not in organic farming. For these CUPs alone, the ratio of residues between conventional and organic increases from 8 to 30. This shows that residues of synthetic CUPs are only a minor issue on soils from organic farms.

Behind these mean aggregated values, however, some cases seem to be hidden, which cannot be explained by “previous applications, drift from conventional fields in the vicinity or atmospheric deposition from distal sources” as stated by Knuth et al.^1^ The possibility of *some* of these residues originating from fraudulent application of synthetic CUPs by organic farmers, however, is not considered by the authors, although it seems to be quite obvious.

For identifying such cases, I did a first screening of the raw data by looking at the percentage of samples with residues above the LOQ (limit of quantification), the mean and the maximum residue concentration for each substance, for organic and conventional farms separately. Whenever the ratio of conventional versus organic was <2 for any of these three indicators, I had a closer look at individual figures. Substances with maximum values of <10 μg kg^–1^ were not further considered because the origin of such low concentrations is often impossible to determine.^4^ Some of the “suspicious” cases detected through this case-by-case analysis are summarized in Table 1.

**Table 1:** Some pesticide residue data extracted from the Supporting Information published by Knuth et al.^1^ and Mark et al.^11^ suggesting that organic farmers may have recently applied the concerned pesticides at their farms

Pesticide	Approval status^a^	No. of positive samples^b^		Mean value (μg kg^–1^)		Maximum value (μg kg^–1^)		Maximum organic value is higher than i-th percentile of conventional^d^		Suspicious cases of organic farms, which may have recently used synthetic pesticides
		Organic	Conventional	Organic	Conventional	Organic^c^	Conventional	positive samples	recorded	
Chlorantraniliprole	Approved	3	12	15.7	30.9	36.3 (IT)	123.4	73rd	98th	One organic sample from Italy with a value of 36.3 μg kg^–1^ is, at least, suspicious.
Chlorpyrifos	Expired January, 10, 2020	21	48	9.7	21.7	100.8 (CH)	210.1	92nd (84th)	no records^e^	There is not much doubt concerning the one organic sample from Switzerland. The percentile in parentheses refers to two samples from Spain with values of 37.6 and 43.3 μg kg^–1^.
Fluopicolide	Approved	3	19	17.9	37.7	30.1 (IT)	120.2	60th (45th)	91st (88th)	The one sample from Italy and the one from France (19.9 mg kg^–1^ percentiles in parentheses) look suspicious.
Prochloraz BTS 44595 (M201-04)	Expired December 31, 2021	2	8	16.7	20.9	20.2 (CZ)	37.3	70th	no records	Two cases from Czech Republic with values of 13.1 and 20.2 μg kg^–1^ are suspicious.
Propamocarb hydrochloride	Approved	1	1	NA	NA	88.5 (IT)	157.7	0th	98th	The one case from Italy seems to be quite obvious.
Tebuconazole	Approved	2	28	177.3	34.5	341.2 (ES)	109.1	100th (27th)	100th (77th)	The one case from Spain is obvious. The percentiles in parentheses refer to asecond case with a value of 13.3 μg kg^–1^, also from Spain.

a Approval status refers to approval in the EU in 2021. Samples for this study were taken in the course of 2021. For chlorpyrifos, this means that farmers could no longer buy it in 2021 but were allowed to use up existing stock until July 10, 2021, while prochloraz could still be used during this year.

b Positive samples means samples with residues greater than the LOQ. The numbers can be considered equivalent to percentages because 101 organic and 100 conventional samples were tested.

c In parentheses the countries, in which the organic soil sample with the maximum values were collected: CH, Switzerland; CZ, Czech Republic; ES, Spain; IT, Italy.

d The maximum organic value is higher than the i-th percentile from conventional farms. Let us take the first line (chlorantraniliprole) as an example: The maximum organic value was 36.3 μg kg^–1^. This is higher than the 73rd percentile of conventional samples, in which residues were found (column "positive"). If we look at all conventional farms, which had **recorded** application of chlorantraniliprole in 2021, then 36.3 μg kg^–1^ is higher than the 98th percentile of the conventional farms because many farms had applied the substance, but no residues were present in soil (column "recorded").

e The database^11^ does not include any application records from 2021 for chlorpyrifos and prochloraz. For chlorpyrifos, this is due to the expiry of approval, while the reason for the prochloraz remains unclear.

If we look, e.g., at chlorpyrifos, this is one of the most widely used insecticides in the world.^5^ The fact that it was found in almost half of all conventional soils in the 2021 study by Knuth et al. shows that also in Europe it was frequently applied before the approval expired in 2020. We have a total of 21 detections on organic farms; 18 of these are in the range between 0.5 and 5.5 μg kg^–1^, which can very likely be explained by soil legacies, spray-drift, or atmospheric transport. There are, however, three suspicious farms: one organic fruit grower in Switzerland with a concentration of 100.8 μg kg^–1^ and two organic vegetable farms in Spain with concentrations of 43.3 and 37.6 μg kg^–1^. These values from Switzerland and Spain are above the 92nd and 84th percentiles, respectively, of conventional farms with residues. Such concentrations most likely stem from recent applications, be it in 2020 or even 2021, not from nonintentional contamination, especially when we consider that insecticide use is common in both fruit and vegetable production.

Asa second example, we can look at tebuconazole. This is a common fungicide effective against Ascomycota, Basidiomycota, and Deuteromycota on a wide range of crops. Residues were found in 28% of conventional soils, but in only 2% of organic soils. This pattern suggests that tebuconazole is far less ubiquitous than, e.g., chlorpyrifos or AMPA (the latter being found in 26% of organic farm soils). What is really striking is the fact that the highest of *all* tebuconazole concentrations was detected on an organic vegetable farm in Spain. There is little doubt that the fungicide had been recently applied on this farm. The second case, with a concentration of 13.3 μg kg^–1^, also from a Spanish vegetable farm, is above the 77th percentile of the conventional farms that had recorded application of tebuconazole in 2021, which should, at least, raise suspicion.

One could argue that apart from these cases of organic farms with relatively high residue levels, there are also many conventional farms on which high residue concentrations were found, contrasting with the fact that no applications of the concerned active ingredients were recorded for 2021. This argument has two weak points. (i) No records for 2021 could mean the substance was applied in 2020. It makes an obvious difference if a pesticide had last been applied one year before sampling, as could be the case for the conventional farms, or five years before sampling, as claimed for the organic farms. (ii) For conventional farmers, too, there may be reasons for incomplete recording of pesticide applications. Many of them, especially in the fruit and vegetable industry, are certified by GLOBALG.A.P.^6^ When a farmer applies pesticides in a way that is not in line with established preharvest intervals or total recommended rates per hectare and season, such applications are likely not to be recorded. In addition, records may be incomplete because of a lack of time or because farmers dislike paperwork. In a survey, 15% of Irish farmers declared “poor compliance” in what refers to recordkeeping of pesticide use.^7^ The real percentage might be higher than reflected in this self-assessment.

There are, thus, a *few* cases of organic farms where recent fraudulent use of synthetic CUPs is quite obvious, in addition to an unknown number of cases that are suspicious in this regard. When Knuth et al.^1^ classify these cases under “previous application, drift or atmospheric transport”, this may have consequences beyond the academic sphere. Research confirming the ubiquity of pesticides in the environment is sometimes used by actors in the organic industry to justify the presence of pesticide residues in organic food,^8–10^ without clearly differentiating between unavoidable contamination and deliberate fraud. This may also happen with the study by Knuth et al. Stakeholders in the organic industry, including some certification bodies, may argue “Science has proven that very high pesticide residues are present in soils even five years after the last application; therefore, it is perfectly plausible that such residues are present in organic food, too”. This reasoning leads to fraudulent applications remaining undetected, which in turn would lead to similar concentrations of pesticides being found in soils from organic farms also in the future; i.e., the misinterpretation of results could have a self-confirming effect.
